# Inhibition of Myeloid Differentiation Factor 88 Reduces Human and Mouse T-Cell Interleukin-17 and IFNγ Production and Ameliorates Experimental Autoimmune Encephalomyelitis Induced in Mice

**DOI:** 10.3389/fimmu.2017.00615

**Published:** 2017-05-29

**Authors:** Shira Dishon, Shmuel J. Cohen, Irun R. Cohen, Gabriel Nussbaum

**Affiliations:** ^1^Institute of Dental Sciences, Hebrew University-Hadassah Faculty of Dental Medicine, Jerusalem, Israel; ^2^Department of Immunology, Weizmann Institute of Science, Rehovot, Israel

**Keywords:** myeloid differentiation factor 88, mixed lymphocyte reaction, experimental autoimmune encephalomyelitis, multiple sclerosis, Th1/Th2

## Abstract

Myeloid differentiation factor 88 (MyD88) recruits signaling proteins to the intracellular domain of receptors belonging to the toll-like/interleukin-1 (IL-1) receptor superfamily. Mice lacking MyD88 are highly susceptible to infectious diseases, but tend to resist experimentally induced autoimmune diseases such as experimental autoimmune encephalomyelitis (EAE) and manifest diminished allograft rejection. We reasoned that inhibition of MyD88 should influence the cytokine profile of responding T cells by blocking costimulatory molecule expression by antigen-presenting cells (APCs) and by inhibiting T-cell responses to IL-18. We now report that inhibition of MyD88 in human APCs led to decreased IFNγ and IL-17 production and a shift to IL-4 production by responding T cells in a mixed lymphocyte reaction. Direct inhibition of Myd88 in mouse and human T cells also reduced their production of IFNγ in response to IL-12/IL-18 stimulation. Finally, systemic MyD88 antagonism significantly reduced the clinical manifestations of EAE in mice. Thus, MyD88 appears to be a key factor in determining T cell phenotype and represents a potential target for therapeutic intervention.

## Introduction

The cytokine environment present during T-cell priming and the costimulatory molecules expressed by antigen-presenting cells (APCs) play a determining role in CD4 T-cell functional differentiation (1). Dendritic cells (DCs) activated by toll-like receptor (TLR) ligands induce T-cell clonal expansion and lineage commitment through the production of cytokines such as interleukin (IL)-12 and IFNγ (2). Myeloid differentiation factor 88 (MyD88) is an adaptor protein shared by TLRs and receptors for the proinflammatory cytokines IL-1 and IL-18. MyD88-dependent signals are critical to the development of Th1 and Th17 responses to foreign and self-antigens, both through the production of cytokines that induce differentiation and factors that suppress Th2 cell development (3, 4). In T cells, MyD88 is also expected to be critical for the response to IL-1 and for IFNγ production in response to IL-18. The net result of these effects is that CD4 Th responses in the absence of MyD88 are strongly skewed to production of Th2 and Tr1 cytokines (5–8).

We previously reported that TLR2 and TLR4 alone did not significantly contribute to experimental autoimmune encephalomyelitis (EAE), however MyD88 was required for EAE induction by active immunization with myelin antigen in Complete Freund’s Adjuvant (CFA); MyD88-deficient mice immunized with MOG35-55 developed a population of IL-10-producing T cells that prevented EAE induced by adoptive transfer of encephalitogenic T cells. These cells also ameliorated EAE when transferred to wild-type mice undergoing active EAE (6). Given its central role in signal-transduction pathways that promote inflammatory Th1 development, MyD88 is an attractive target for therapeutic interventions in autoinflammatory and autoimmune diseases. MyD88 is composed of an N-terminal death domain and a C-terminal Toll/IL-1 receptor (TIR) domain separated by a short intermediary sequence (9). Following activation, MyD88 dimerizes and assembles a multiprotein complex that enables recruitment of the IL-1 receptor-associated kinases (IRAK)-4 and IRAK-1 and phosphorylation of TRAF6 (10, 11). MyD88 dimerization is dependent on a region within the TIR domain known as the BB loop that connects the second β-strand to the second α-helix. A heptapeptide from this region competitively inhibits MyD88 dimerization and inhibits TLR/IL-1R signaling *in vitro* and *in vivo* (10, 12–14).

Herein, we determined the role of MyD88 in human T-cell phenotype modulation by inhibiting MyD88 in either the APCs or the T cells. Silencing MyD88 in APCs shut off IFNγ and IL-17 production by alloreactive human CD4 T cells and shifted the response to IL-4 production. Targeting T-cell MyD88 blocked the response to IL-12/IL-18 stimulation. Furthermore, MyD88 inhibition *in vivo* in mouse EAE shifted the T-cell cytokine profile and led to reduced clinical signs of disease.

## Materials and Methods

### Cell Culture

U-937, THP-1, and HeLa human cell lines (ATCC), and human and murine primary cells, were grown in RPMI or DMEM (Sigma) supplemented with fetal calf serum (FCS, 10%), 4 mM l-glutamine, 1 mM sodium pyruvate, penicillin (100 U/mL), and streptomycin (0.1 mg/mL) (Biological Industries, Israel) at 37°C and 5% CO_2_. U-937 and THP-1 monocytes were differentiated to macrophages with 5 ng/mL PMA (Cayman Chemical, Ann Arbor, MI, USA) for 3 days. Primary human CD4^+^ T cells and monocytes were purified from buffy coats by the RosetteSep Human CD4^+^ T or the RosetteSep human monocyte cell enrichment cocktail according to the manufacturer’s instructions (StemCell Technologies, USA). Primary monocytes were differentiated to macrophages (confirmed by morphology and adhesion) with 10 ng/mL GM-CSF (Peprotec, Rehovot, Israel) for 6 days.

### Lentiviral Transduction of shRNA

Five different MyD88 shRNA lentiviral transduction particles (Sigma) were independently transduced, and stable clones (of THP-1, U-937, and HeLa cells) were obtained based on resistance to puromycin (Gold Biotechnology. St. Louis, MO, USA). The shRNA sequence that reduced human MyD88 to the greatest extent was 5′-CCGGCCTGTCTCTGTTCTTGAACGTCTCGAGACGTTCAAGAACAGAGACAGGTTTTTG-3′. Non-target shRNA control cells were generated using an irrelevant sequence (Sigma). To transduce, cells were plated at 75% confluency 3 h prior to transduction and then the corresponding lentiviral particles were added at a Multiplicity of Infection of 10 overnight. After 48 h, cells were selected by addition of medium containing 2 µg/mL puromycin for THP-1 cells and 0.5 µg/mL for U-937 and Hela cells.

### mRNA Isolation and Reverse Transcription qPCR

Total cellular RNA of the transduced cell lines was extracted using tri-reagent (Sigma) and the mRNA was reverse transcribed to cDNA with Maxima H minus Enzyme mix, according to the manufacturer’s instructions (Thermo Scientific, Waltham, MA, USA). Amplification for qPCR was performed with primers to hMyD88 (forward 5′-ACAGGCACCAGCATACAC-3′; reverse 5′-TTGGGTCCTTTCCAGAGT-3′) and hβactin (forward 5′-CACGGCATCGTCACCAACT-3′; reverse 5′-TGATCTGGGTCATGTTCTCGC-3′). qPCR was performed using Bio-Rad CFX manager (Bio-Rad, Hercules, CA, USA) and the reactions with KAPA SYBR Fast ABI prism (KAPA systems, Salt River, Cape Town). The final results were calculated by dividing the relative transcript levels of the test genes by the relative amount of βactin RNA.

### Western Blot

For MyD88 protein detection, 10^7^ cells were lysed in Ripa buffer in the presence of protease inhibitors and incubated on ice for 25 min. The lysates were then centrifuged at 10,000*g* for 15 min. Proteins were separated by gel electrophoresis and transferred to nitrocellulose membranes. The nitrocellulose membranes were blocked with 5% skim milk for 1 h and MyD88 was detected using 1 µg/mL polyclonal rabbit anti-MyD88 (eBioscience, San Diego, CA, USA) followed by 100 ng/mL goat anti-rabbit IgG-HRP (Abcam, Cambridge, UK). After washing extensively with Tris-buffered saline (TBS) containing 0.2% Tween 20, the membranes were exposed to chemiluminescent substrate in the presence of hydrogen peroxide, using the EZ-ECL-chemiluminescence detection kit (Biological Industries, Israel). A Gel-Doc imaging system (Bio-Rad, Hercules, CA, USA) was used to capture the image.

### Flow Cytometry

THP-1 cells were seeded at 1.5 × 10^6^ in six well plates with 5 ng/mL PMA (Cayman Chemical, Ann Arbor, MI, USA) for 3 days differentiation. When indicated, 100 ng/mL Pam3csk4 (Invitrogen, San Diego, CA, USA) + 20 ng/mL IFNγ (Peprotec, Rehovot, Israel) was added to the cells for the final 24 h of incubation to induce differentiation (concentrations of stimulants were determined in preliminary calibration experiments). Cells were then collected, washed, and stained using APC-conjugated human-specific antibodies (Biolegend, San Diego, CA, USA) to CD80, HLA-A2, and HLA-DR for 30 min on ice. The purity of CD4^+^ T cells isolated from buffy coats of healthy donors was confirmed by flow cytometry using FITC-anti-CD4 (Tonbo Biosciences, San Diego, CA, USA). Intracellular cytokine staining was analyzed on T cells incubated for 6 days with APCs. Cells were incubated with cell stimulation cocktail (eBioscience, San Diego, CA, USA) and Brefeldin (Biolegend, San Diego, CA, USA) for 6 h prior to collection, and then fixed 15 min at RT. Cells were then permeabilized and stained with Leucoperm permibilization reagent (AbD Serotec, Kidlington, UK) and APC-anti-IL-4 (Biolegend, San Diego, CA, USA) for 30 min on ice. Samples were analyzed using a BD LSRII flow cytometer or a BD ACCURI C6 cytometer (BD Biosciences, San Jose, CA, USA), and data were analyzed using FCS Express software (*De Novo* Software, Glendale, CA, USA).

### Analysis of p65 Nuclear Translocation

Hela shCnt and HeLa shMyD88 cells were activated with 20 ng/mL IL-1β or 10 ng/mL TNFα (ProSpec, Rehovot, Israel) for 30 min or 1 h, respectively. Cells were then fixed (3.7% PFA in PBS for 10 min), permeabilized (0.25% Triton-X100) and blocked (2% BSA in TBS) at 4°C for 16 h. Cells were then stained with 0.6 µg/mL rabbit anti-p65 in 2% BSA in TBS (Santa Cruz Biotechnology, Dallas, TX, USA) followed by 0.5 µg/mL CY-3 goat anti-rabbit antibody (Jackson ImmunoResearch, Baltimore Pike, PA, USA). Cytoplasmic vs. nuclear localization was analyzed by fluorescent microscopy (15) (Nikon-Ti microscope).

### Peptides

Myeloid differentiation factor 88 inhibitor peptide (MyDI) (RDVLPGT), or the scrambled version of the same sequence, (MyDI-sc) (PTDLVRG), were synthesized in the Weizmann Institute of Science and purified by HPLC.

### Mixed Lymphocyte Reaction (MLR)

shMyD88 THP-1 vs. shCnt THP-1 cells were differentiated as above with PMA alone or PMA with Pam3csk4/IFNγ. Cells were treated with 10 µg/mL Mitomycin c (Sigma) to prevent replication, and added in various concentrations to CD4^+^ T cells in 96 well round bottom plates (Thermo Scientific). T-cell proliferation and cytokine production were analyzed after 6 days of coculture. Proliferation was assayed using a non-radioactive Tetrazolium-based kit (Promega, Madison, WI, USA) according to the manufacturer’s instructions.

Primary human macrophages were incubated for 24 h with or without 80 µM MyDI vs. MyDI-sc peptides. Macrophages were treated with 10 µg/mL mitomycin c (Sigma) to prevent replication and added in various concentrations to CD4^+^ T cells in 96-well round bottom plates (Thermo Scientific). T-cell proliferation and cytokine production were analyzed after 6 days of coculture. Proliferation was assayed using a non-radioactive Tetrazolium-based kit (Promega, Madison, WI, USA) according to the manufacturer’s instructions.

### MyD88 Inhibition of Mouse MOG-Specific T Cells and Human CD4^+^ T Cells

Anti-MOG35-55 resting T cells were treated *in vitro* with MyDI or MyDI-sc (20 µM) prior to stimulation with IL-18 (30 ng/mL) (R&D systems, Inc., Minneapolis, MN, USA). The effect of MyDI or MyDI-sc was also tested on anti-MOG35-55 resting T cells re-stimulated with MOG35-55 (5 µg/mL) in the presence of irradiated splenocytes with or without the addition of IL-18. Cytokines were measured in the cell supernatants after overnight incubation. Primary human CD4^+^ T cells that were isolated from buffy coats were incubated for 3 h in the absence or presence of MyDI, or MyDI-sc (20 µM) and stimulated with IL-18 + IL-12 (320 and 160 ng/mL, respectively) overnight (Peprotec, Rehovot, Israel). Cytokines were measured in the cell supernatants after overnight incubation.

### Mice

C57BL/6 (B6) mice were purchased from Harlan (Jerusalem, Israel). Female, 8- to 14-week-old mice were used in the experiments. The mice were housed at the SPF units of our universities and all experiments were approved by the Hebrew University-Hadassah Institutional Animal Care and Use Committee (protocol number 16-14745).

### Draining Lymph Node (DLN) Cell Activation

Mice immunized s.c. with 100 µg MOG_35-55_/CFA and treated with MyDI and control were sacrificed 11 days after immunization, and popliteal, inguinal, and axillary LNs were collected and single cell suspensions were prepared. Cells were cultured in 96-well plates (0.5 × 10^6^ per well) for 72 h with or without increasing concentrations of Myelin Oligodendrocyte Glycoprotein (MOG_35-55_) or purified protein derivative (PPD) or ovalbumin (OVA) peptide in the culture.

### Induction of EAE and Treatment

For *in vivo* treatment, we synthesized the MyDI or MyDI-scr peptides with a basic peptide from the Drosophila Antennapedia homeodomain (DRQIKIWFQNRRMKWKK) at the C-terminus [12]. All peptides were synthesized at the Weizmann Institute of Science and purified by HPLC. Mice were immunized s.c. in the flank with 200 µg MOG_35-55_ emulsified in CFA supplemented with 300 µg Mycobacterium *tuberculosis* (Mt) H37RA (Difco). Pertussis Toxin (PTX, List Biological Laboratories, CA, USA) was injected i.v. at the time of immunization and 48 h later. In some experiments, animals were sacrificed prior to onset of clinical symptoms in order to analyze the lymph node response. EAE was scored on a scale of 0–6: 0, no impairment; 1, limp tail; 2, limp tail and hind limb paresis; 3, ≥1 hind limb paralysis; 4, full hind limb and hind body paralysis; 5, hind body paralysis and front limb paresis; and 6, death. Mice were treated with MyDI i.p. (2 mg/kg) vs. control at day 0 and every 48 h.

### Cytokine Analysis

Levels of hTNF-α, h INF-γ and hIL-17 and mIL-10, mIL-5, mIL-17, and mINF-γ were determined using human/mouse OptEIA sets (BD Biosciences, CA, USA) according to the manufacturer’s instructions.

### Statistical Analysis

The two-tailed *t*-test was used for statistical evaluation of all the results except the two-way analysis of variance test that was used for the EAE model assay. Values are shown for data that reached a significance of *P* ≤ 0.05 (*), *P* ≤ 0.01 (**), *P* ≤ 0.005, (***), and *P* ≤ 0.001 (****). Bars show mean and SD and in Figure 6D SEM (Prism v.5, GraphPad Software Inc., San Diego, CA, USA).

## Results

### Silencing MyD88 with shRNA Specifically Blocks the Response of Human Cell Lines to TLR/IL-1 Stimulation

**Figure 1:**
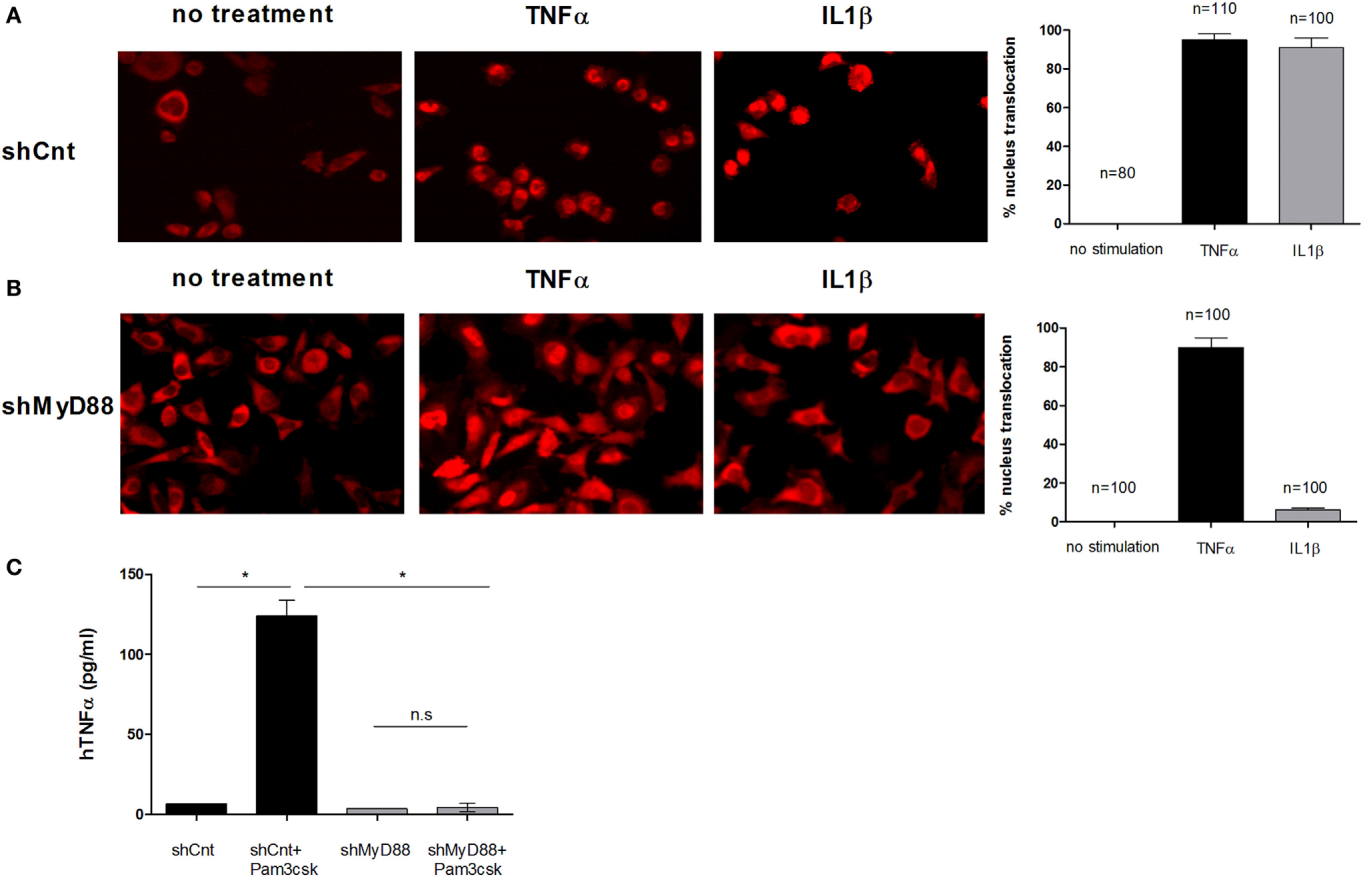
**Inhibition of myeloid differentiation factor 88 (MyD88) blocks NFκB p65 translocation and hTNFα production**. Control shRNA **(A)** or shMyD88 **(B)** lentivirally transduced HeLa cells were stimulated with recombinant hTNFα (black bars) or hIL-1β (gray bars) and NFκB localization was determined using anti-p65 conjugated to rhodamine. The bar graphs represent the percent of cells with nuclear p65 localization. The percentage of cells with nuclear p65 was determined in three independent experiments and the SDs between experiments is indicated by the error bars. The number of cells quantified is indicated. **(C)** THP-1 cells were differentiated and stimulated with the Toll-like receptor (TLR)2 ligand Pam3csK4 (200 pg/mL) overnight. TNFα levels in the supernatants were determined by ELISA. One representative experiment of five independent experiments performed in triplicate, is shown. The two-tailed *t*-test was used for statistical evaluation.

To determine the role of MyD88 in human APCs, we first established stable shRNA knock-down cells. We screened lentivirally expressed shRNA sequences in HeLa cells to find a sequence that reduced MyD88 efficiently and did not affect inflammatory pathways driven by other signals. Sequences that reduced MyD88 mRNA expression also blocked NFκB nuclear translocation in response to IL-1 stimulation, a MyD88-dependent event, but had no effect on NFκB translocation in response to TNFα stimulation that occurs independently of MyD88 (Figure 1B). Control shRNA transduction did not affect either signaling pathway (Figure 1A). We next transduced human THP-1 monocyte/macrophage cells with this shRNA and found efficient reduction of MyD88 mRNA and protein (Figure S1 in Supplementary Material), and a near complete reduction in the response to stimulation with PAM3Csk4, a synthetic triacylated lipopeptide ligand of TLR2/TLR1 (Figure 1C).

### MyD88 Silencing Blocks the Induction of Macrophage CD80 But Does Not Affect HLA Expression in Human APCs

**Figure 2:**
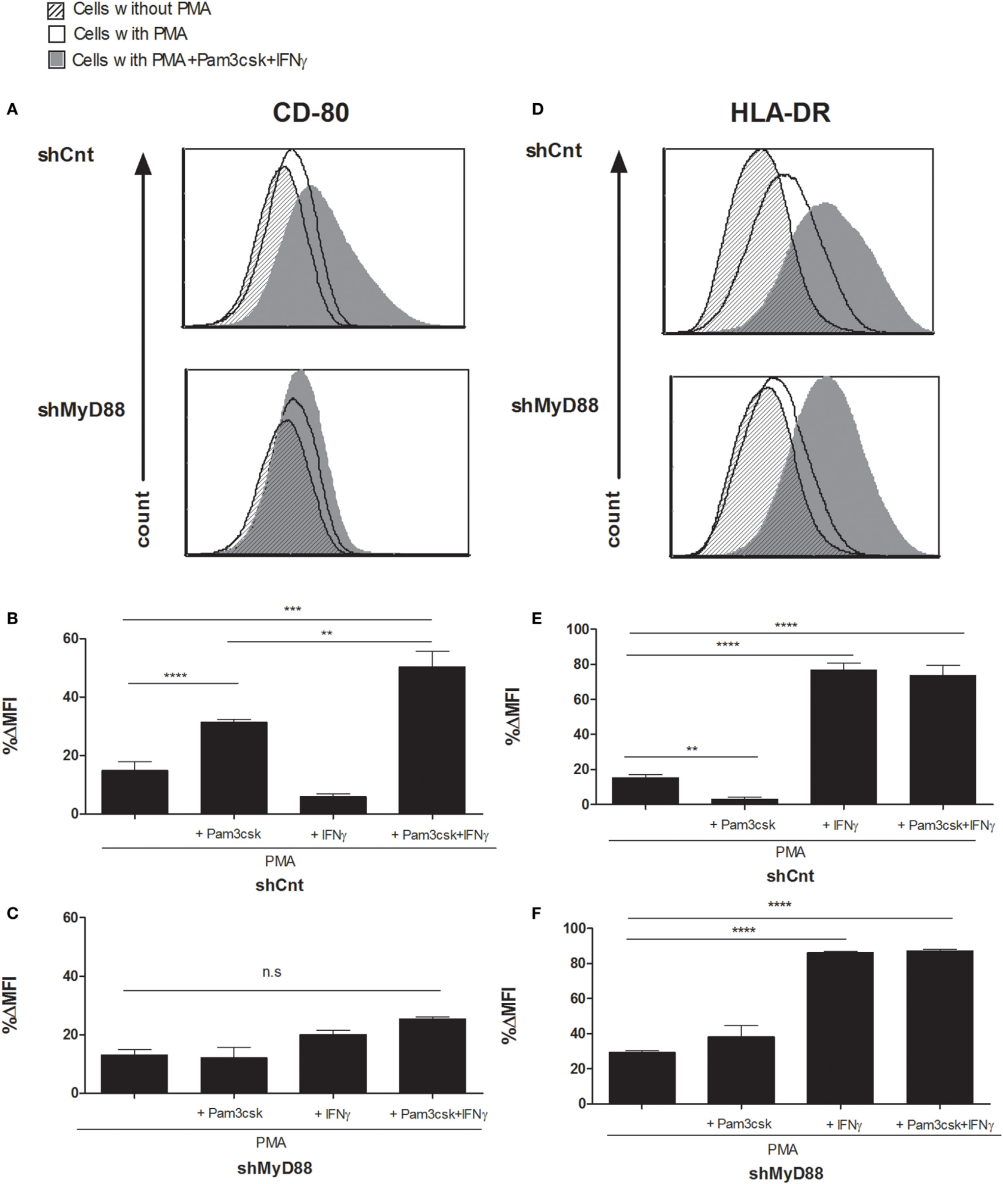
**Myeloid differentiation factor 88 (MyD88) knockdown blocks the induction of macrophage CD80 expression without affecting HLA-DR in response to Toll-like receptor (TLR) ligands**. Control (shCnt) vs. shMyD88 transduced THP-1 cells were differentiated for 72 h with PMA, with the addition of 100 ng/mL Pam3csk4, 20 ng/mL IFNγ, or 100 ng/mL Pam3csk4 + 20 ng/mL IFNγ. For the final 24 h. Cells were then analyzed for CD80 **(A–C)** and HLA-DR **(D–F)** expression by flow cytometry. Data in panels (**B,C,E,F**) are presented as % difference in intensity between each group (ΔMFI). Representative histograms **(A,D)** and combined data of triplicates from two independent experiments are shown (**B,C,E,F**). The two-tailed *t*-test was used for statistical evaluation.

To probe the effect on T-cell phenotype of silencing MyD88 in APCs, we first tested costimulatory molecule (CD80) and HLA expression in shMyD88 compared to shCnt THP-1 cells differentiated with PMA and further stimulated with Pam3csk4/IFNγ. We focused on CD80 since CD86 expression was not strongly upregulated in response to stimulation with Pam3csk4/IFNγ. In shCnt cells, Pam3csk4 alone induces CD80 expression which is further enhanced by addition of IFNγ (Figures 2A,B). Silencing MyD88 prevented the induction of CD80 expression in response to Pam3csk4/IFNγ (Figures 2A,C), since enhanced CD80 expression is mostly due to TLR2 stimulation by PAM3csk4 (Figures 2B,C). However, the induction of HLA expression (both class I and class II), an effect attributable to IFNγ stimulation (Figures 2E,F), remained intact in the absence of MyD88 (Figures 2D–F; Figure S2 in Supplementary Material). Interestingly, Pam3csk4 reduced the basal level of MHC-II expression in differentiated shCnt THP1 cells (Figure 2E); however, this effect was not seen in the WT THP1 cells (not shown). Therefore, MyD88 affects the regulation of costimulatory signals without inhibiting the regulation of HLA.

### MyD88 Knockdown APCs Induce T Cell Proliferation with Enhanced IL-4 Production and Lowered IFNγ and IL-17 Production

**Figure 3:**
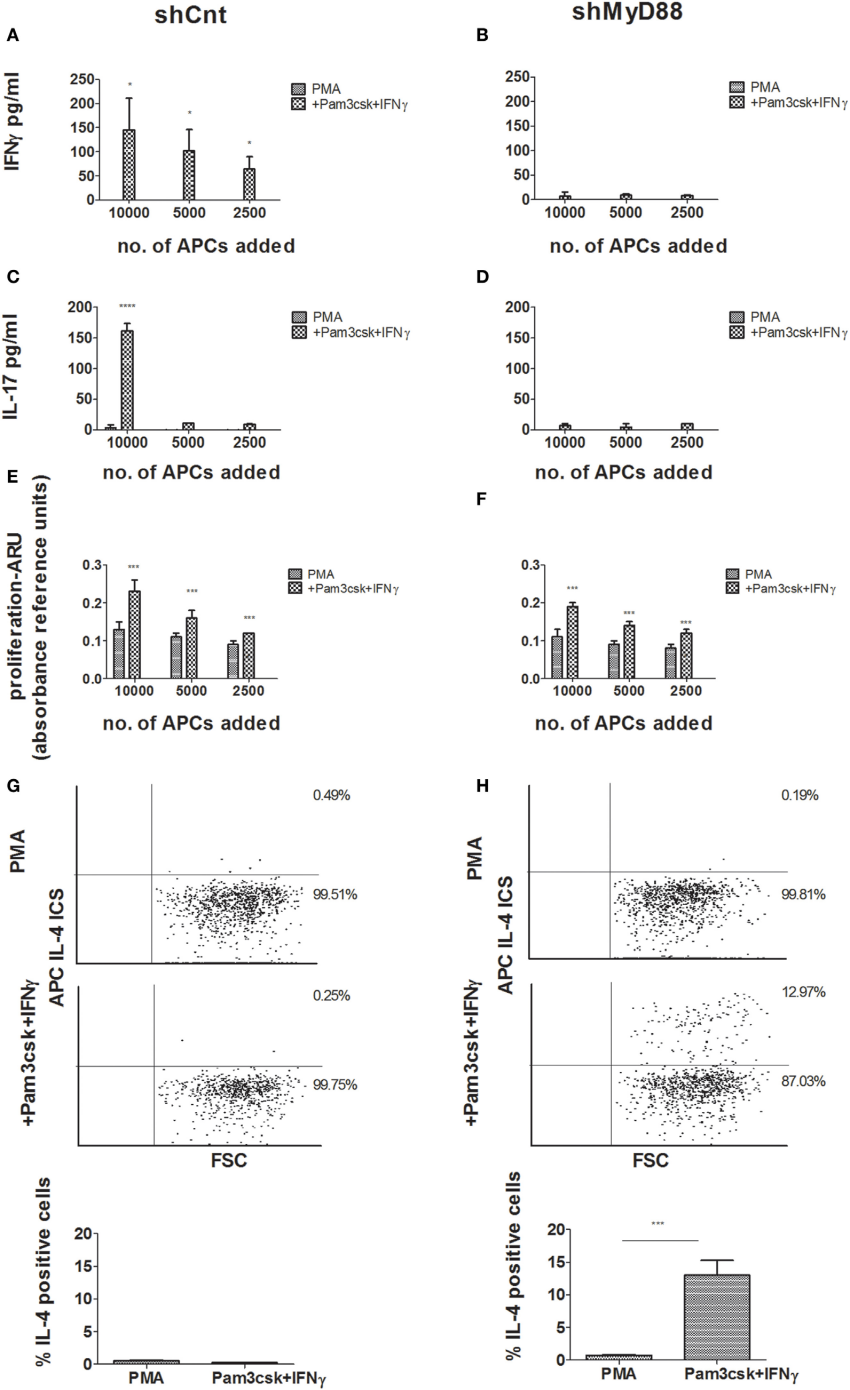
**Mixed lymphocyte reaction (MLR) responses using control vs. shMyD88-transduced antigen-presenting cells**. Primary human CD4^+^ T cells were isolated from buffy coats and activated by incubation with decreasing concentrations of shCnt, or shMyD88 THP-1 cells, previously differentiated with PMA, or PMA with the addition of Pam3csk4 + IFNγ. Cytokine production and proliferation were determined after 6 days of incubation. **(A,B)** IFNγ production, **(C,D)** IL-17 production, and **(E,F)** proliferation. For panels **(A–D)**, one representative experiment of three performed with similar results, is shown. For proliferation **(E,F)**, the combined results of the three independent experiments are shown. In the proliferation studies **(E,F)**, the ARU of T cells without THP-1 cells was close to 0. **(G,H)** Flow cytometry analysis of intracellular IL-4 expression is presented for primary human CD4^+^ T cells stimulated by incubation with shCnt, or shMyD88 THP-1 cells, that were differentiated with PMA, or PMA with the addition Pam3csk4 + IFNγ: **(G)** shCnt and **(H)** shMyD88 cells. One representative experiment of three independent MLR experiments is shown in the dot plots, and the average of the three experiments is shown in the bar graphs. The two-tailed *t*-test was used for statistical comparison.

We next performed a MLR using the shMyD88 compared to shCnt THP-1 cells. The THP-1- transfected cells were differentiated for 3 days, and during the final 24 h the cells were stimulated with Pam3csk4/IFNγ. The THP-1 cells were treated with Mitomycin c to prevent their proliferation, and then seeded together with CD4^+^ cells isolated from buffy coats. After 6 days, we measured T-cell proliferation and cytokine production. ShCnt and shMyD88 APCs induced T-cell proliferation to a similar extent (Figures 3E,F, no significant differences were detected between the proliferation induced by each type of APC). Nevertheless, there was a marked difference in the cytokine profile of the proliferating T cells. Whereas shCnt APCs induced IFNγ (Figure 3A) and IL-17 (Figure 3C) in a concentration-dependent manner, there was no production of either of these cytokines by T cells stimulated with shMyD88 APCs (Figures 3B,D). In contrast, the shMyD88 APCs induced IL-4-producing T cells, which were not present in the cultures of cells stimulated with shCnt APCs (Figures 3G,H).

**Figure 4:**
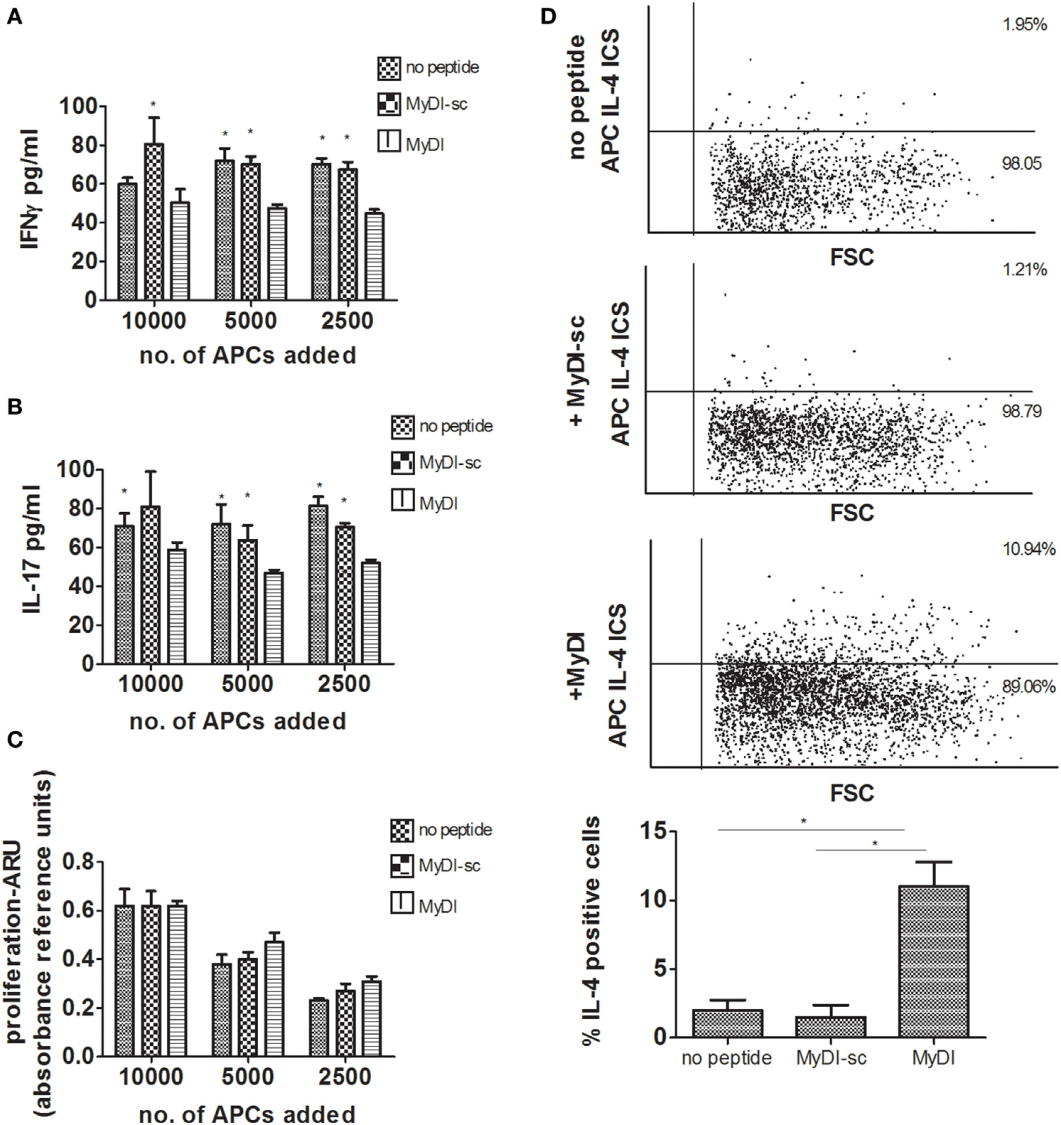
**Peptide inhibition of myeloid differentiation factor 88 in primary human antigen-presenting cells (APCs) leads to a shift in cytokine production by responding T cells**. Primary monocytes were isolated from buffy coats and differentiated to macrophages for 6 days. Donor mismatched human CD4^+^ T cells were isolated from buffy coats and activated by incubation with decreasing concentrations of macrophages pretreated with or without MyDI/MyDI-sc (80 µM). After 6 days of incubation, production of IFNγ **(A)** and IL-17 **(B)**, and proliferation **(C)**, were determined. Asterisks represent statistical comparison between the specified group vs. treatment with the MyDI peptide. For panels **(A,B)**, one representative experiment of three independent experiments is shown. In panel **(C)**, the proliferation of the three experiments was combined. Intracellular IL-4 expression was determined by flow cytometry **(D)**. One representative experiment of three independent mixed lymphocyte reaction (MLR) experiments is shown in the dot plots, and the average of the three experiments is shown in the bar graphs. The two-tailed *t*-test was used for statistical evaluation.

To strengthen the results, we next examined the effect of MyD88 inhibition using primary human APCs and an additional means of MyD88 inhibition. To block MyD88, we incubated differentiated primary macrophages with the MyD88 inhibitory BB loop peptide (MyDI) (12), and as a control we used a scrambled control peptide (MyDI-sc). MyDI treatment specifically blocks MyD88-dependent signaling, as demonstrated by reduced NFκB activation in response to IL-1, but not in response to TNFα stimulation. In contrast, MyDI-sc does not inhibit NFκB activation in response to either stimulant (Figure S4 in Supplementary Material). MyDI treatment also blocks inflammatory cytokine production by primary cells or THP-1 cells activated with the TLR2 ligand Pam3csk4, although to a lesser extent than shRNA inhibition (Figures S5A,B in Supplementary Material). Primary APCs were treated with Mitomycin c to prevent their proliferation, and then seeded together with donor-mismatched CD4^+^ T cells isolated from buffy coats. After 6 days, we measured T-cell proliferation and cytokine production. Inhibition of MyD88 did not affect the ability of primary APCs to induce T cell proliferation (Figure 4C), however inhibition shifted the cytokine profile of the proliferating T cells. Similar to our findings using shMyD88 THP-1 cells, peptide inhibition of MyD88 in primary APCs led to significantly diminished IFNγ and IL-17 by responding T cells, and a shift to IL-4 production (Figures 4A,B,D). Importantly, peptide blocking of MyD88, a less efficient method compared to stable shRNA inhibition, produced a similar effect. Similar results were obtained when primary human DCs were used as APCs (data not shown). Therefore, MyD88 inhibition in human APCs induces a shift in the cytokine profile of responding T cells from IFNγ and IL-17 production to IL-4 production without significantly reducing T-cell proliferation.

### MyD88 Inhibitor Peptide Reduces IFNγ Secreted by IL-18 ± IL-12 Stimulated Human and Mouse T-Cells

**Figure 5:**
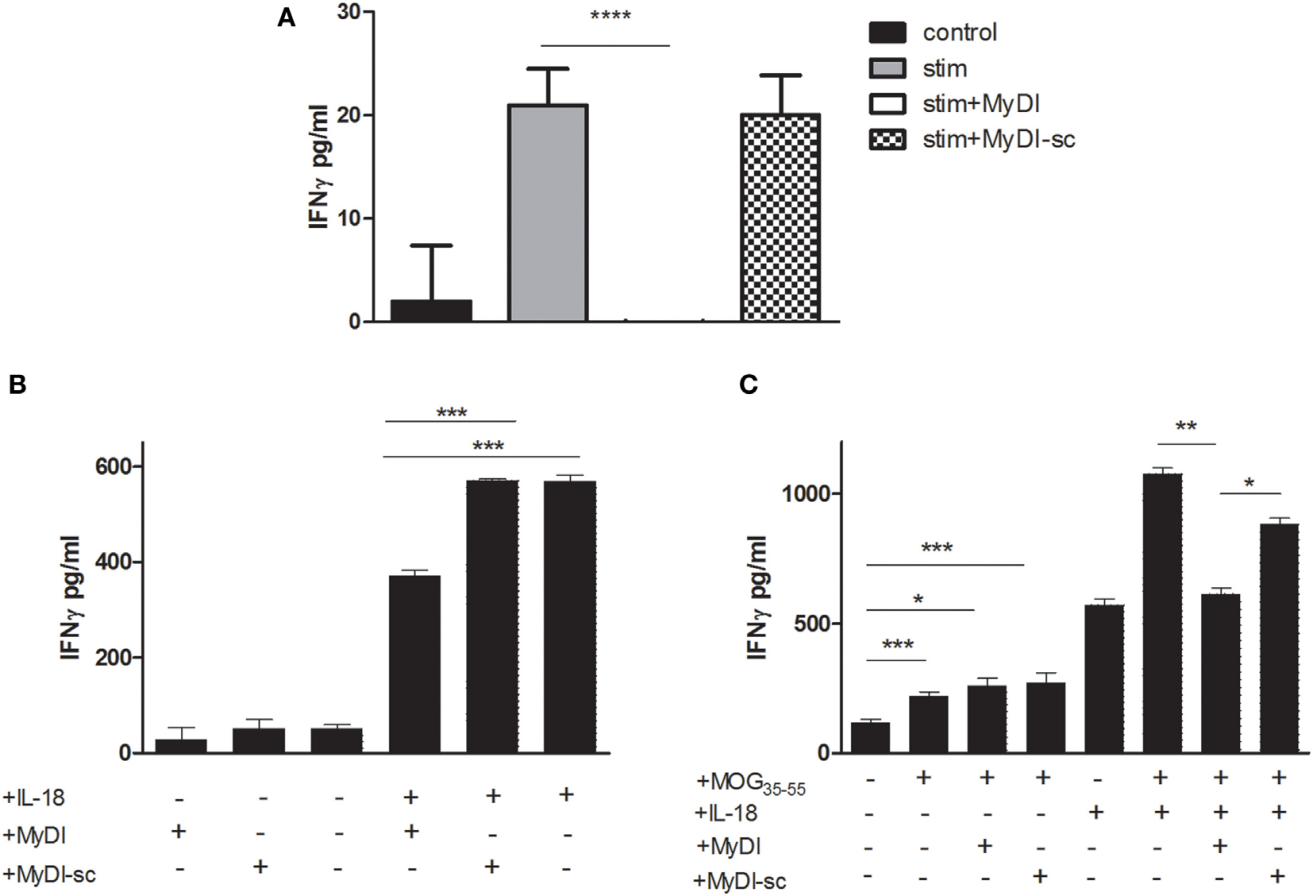
**Inhibition of myeloid differentiation factor 88 in murine and human T cells reduces IFNγ production**. **(A)** Primary human CD4^+^ T cells were isolated from buffy coats and stimulated with IL-18 + IL-12 (320 and 160 ng/mL, respectively) overnight in the absence or presence of MyDI or MyDI-sc (20 µM/mL). The results are the average of triplicates and one representitve experiment of three independent experiments is shown. **(B)** Murine MOG35-55 specific T cells were incubated with MyDI or MyDI-sc (20 µM/mL) or without peptide for 2 h. T-cells were then plated with or without 30 ng/mL IL-18 ON and IFN-γ production was measured by ELISA. **(C)** MOG35-55 specific T cells were incubated with MyDI or MyDI-sc, or without peptide for 2 h. T-cells were then plated with or without 30 ng/mL IL-18 ON in the presence of irradiated splenocytes and MOG35-55 peptide (5 µg/mL). The results are average of triplicates and representitve of two independent experiments. The two-tailed *t*-test was used for statistical evaluation.

Since MyD88 functions downstream to multiple receptors expressed by T cells, we next asked if inhibition of T-cell MyD88 would effectively reduce cytokine production. In contrast to THP-1 cells, we found that the MyDI peptide penetrated primary T cells efficiently (Figure S3 in Supplementary Material), obviating the need for shRNA. To investigate the effect of MyD88 inhibition on primary human T cell antigen-independent IFNγ production (16), cells were incubated with MyDI or MyDI-sc and then stimulated overnight with IL-18 + IL-12. hIFNγ secretion was abolished in the stimulated cells by the addition of MyDI but was not decreased in the cells treated with the MyDI-sc (Figure 5A). To test if similar effects could be obtained in autoimmune T cells, we treated MOG_35-55_-specific murine T cells with MyDI and MyDI-sc prior to IL-18 stimulation. MyDI treatment significantly reduced IL-18-stimulated IFNγ secretion, in contrast to treatment with MyDI-sc (Figure 5B). We next tested the effect of MyDI on the cytokine response of MOG_35-55_-specific T cells responding to antigen stimulation in the presence of IL-18 and MOG_35-55_ peptide (Figure 5C). IL-18 treatment increased the IFNγ response of antigen-specific T cells. Here too, MyDI treatment significantly reduced the level of IFNγ when IL-18 was present (Figure 5C).

### MyD88 Inhibitor Peptide Alters the Cytokine Profile of the Inflammatory Response *In Vivo* and Attenuates EAE

**Figure 6:**
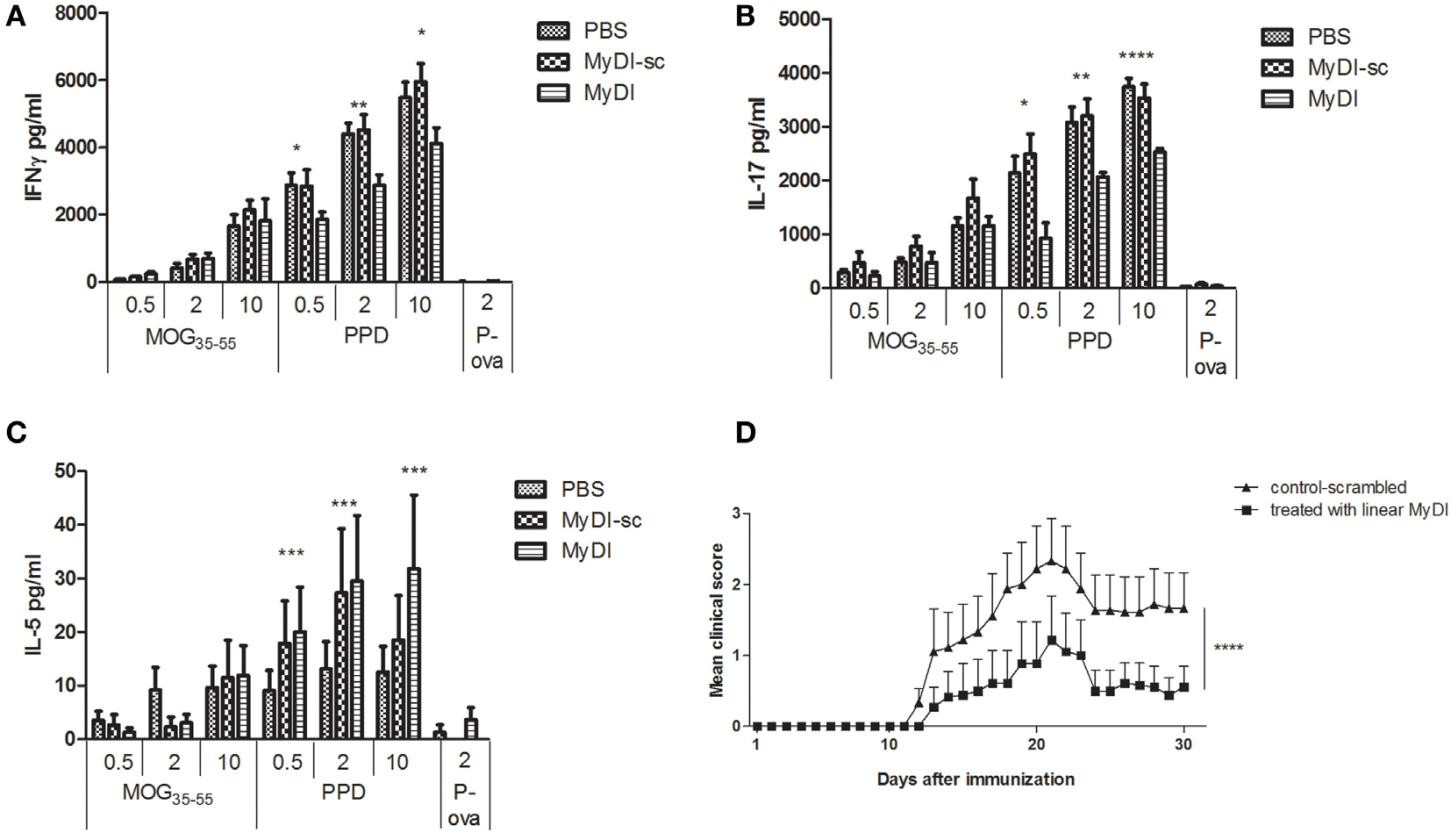
**MyDI administration *in vivo* alters the cytokine profile of responding T cells and ameliorates severity of experimental autoimmune encephalomyelitis (EAE)**. LNC were harvested 11 days after MOG35-55/CFA immunization. Groups were treated at day 0 and every 48 h i.p. with 2 mg/kg MydI, or MyDI-scrambled or PBS as controls. Cells were plated and activated *ex vivo* with increasing doses of MOG35-55 or increasing doses of purified protein derivative (PPD; 0.5, 2, and 10 µg/mL), or ovalbumin (OVA) peptide (2 µg/mL), an irrelevant antigen control. Cytokines were measured in the supernatants at 72 h. LNC from animals treated with MyDI produced significantly less IFN-γ **(A)** and IL-17 **(B)**, but significantly more IL-5 **(C)**, at all concentrations of PPD antigen. **(A–C)** Results are the average of triplicates and the two-tailed *t*-test was used for statistical evaluation. **(D)** Mice were immunized with MOG35-55/CFA on day 0, with PTX administration on days 0 and 2. Groups of mice were treated with 2 mg/kg MyDI (squares) or MyDI-sc/PBS (triangles). The graph shows differences in clinical scores between mice treated with MyDI and controls (*n* = 9 mice per group). Data are mean ± SEM and significantly different by two-way analysis of variance (ANOVA).

To investigate the *in vivo* effects of MyDI treatment on the T cell cytokine profile, we immunized mice with MOG_35-55_ peptide emulsified in CFA and administered PTX on day 0 and at 48 h. The mice were treated i.p. on day 0 and every 48 h with 2 mg/kg MyDI or MyDI-sc or PBS. Eleven days following immunization, the DLNs were harvested and cells were activated *ex vivo* with increasing doses of MOG_35-55_ or increasing doses of PPD, representing major antigens of the Mtb in the adjuvant. Cytokine secretion was analyzed 72 h after activation. As shown in Figure 6, MyDI treatment induced a shift in the cytokine profile of the LN T cell response to PPD. T cells from MyDI-treated animals secreted significantly less IFNγ and IL-17 (Figures 6A,B), but greater amounts of IL-5 (Figure 6C). Since MyDI treatment influenced the T cell cytokine profile, we next tested the effect of MyDI administration on the clinical outcome of MOG_35-55_/CFA-induced EAE. Mice were treated with MyDI or MyDI-sc (2 mg/kg) or PBS (200 µL) i.p. three times a week beginning on the day of immunization with MOG_35-55_/CFA. As shown in Figure 6D, MyDI treatment significantly reduced EAE disease severity.

## Discussion

Myeloid differentiation factor 88 is an adaptor protein that assembles a signaling complex at the intracellular domains of TLRs and the IL-1R superfamily, leading to the downstream activation of NF-κB and MAPKs (11, 17, 18). MyD88-dependent signals, in both APCs and responding T cells, play a major role in determining the strength and character of T cell responses (19). MyD88-deficient mice are for the most part resistant to autoimmune disease, and are unable to reject minor- and major-mismatched allograft transplants (4, 20–22). However, we know less about the role of MyD88 in determining the outcome of human T cell responses, and clinical features of MyD88 deficiency in humans do not mimic the critical role played by MyD88 in mice. For example, MyD88-deficiency in humans enhances the sensitivity to infection by a narrow range of mostly pyogenic bacteria, in contrast to the broad-spectrum risk of infection in MyD88 knockout mice (23–26). Furthermore, the sensitivity to infection in individuals with inactivating mutations in MyD88 wanes with age, suggesting that MyD88 becomes redundant in the adult human response to infection. In this study, we explored the role of MyD88 in regulating the outcome of a human alloreactive MLR response. MyD88 knockdown human APCs expressed equivalent levels of MHC but decreased CD80 compared to control APCs in response to TLR2-induced maturation (together with IFNγ). MyD88 inhibition in human APCs (either a cell line or primary cells) did not affect the ability of the APCs to induce proliferation of alloreactive T cells in an MLR; however, the cytokine profile shifted from IFNγ/IL-17 production, to IL-4 production. Reduced CD80 expression (without changes in CD86 expression) has been shown by others to correlate with a shift in the T cell response from IFNγ production to IL-4 production, and with enhanced allograft survival (27). Inhibition of MyD88 in human APCs produced a similar outcome to that of alloreactive responses in mouse T-cells stimulated by DCs from MyD88-deficient mice (5), and our results are consistent with the shift to Th2-cytokines observed in multiple immunization studies using MyD88-deficient mice (28–30). In contrast, others have shown that the TRIF signaling pathway, rather than the MyD88 pathway, induces upregulated costimulatory molecule expression in mouse (31) or human (32) DCs. Our study ruled out TRIF involvement by differentiating APCs in the presence of the TLR2 ligand PAM3Csk4; however, we also found that MyD88, rather than TRIF, was responsible for costimulatory molecule expression and T-cell Th1-differentiation when LPS was used to differentiate the APCs (data not shown). To the best of our knowledge, this is the first demonstration of the role of MyD88 in a human alloreactive response, and our results indicate that MyD88 inhibition is sufficient to shift the phenotype of the T cell response to alloantigens.

Myeloid differentiation factor 88 signaling also controls T-cell responses intrinsically, both because of its role in the signaling of IL-1R family members expressed on T cells and because T cells express innate immune TLRs and respond to TLR ligands (33–35). In mouse T cells, intrinsic MyD88-signaling is essential for both early differentiation of Th17 cells (36) and for Th17 lineage commitment (37). IL-1β, together with IL-6, induces Th17 polarization of naïve human CD4^+^ T cells independent of antigen stimulation (38). We found that MyD88 inhibition in human CD4^+^ T cells blocked IFNγ production in response to IL-12 + IL-18 stimulation, confirming that MyD88 is not redundant for human T-cell responses to IL-1 family members. Similarly, MyD88 inhibition of mouse T cells *in vitro* reduced their response to IL-18. Thus, in the setting of antigen presentation, interventions that block MyD88-signaling can alter the character of the adaptive response by affecting the APCs, and/or the responder T cells directly. Given its role in determining T-cell phenotype, targeted inhibition of MyD88-mediated signaling represents an appealing therapeutic strategy for organ transplantation and autoimmune disease. Importantly, intra-peritoneal administration of the MyD88 inhibitory peptide to mice immunized with MOG/CFA was sufficient to significantly shift the cytokine response of lymph node T cells toward greater IL-5 production and less IFNγ/IL-17 production. The cytokine shift observed in mice treated with the MyD88-inhibitor peptide correlated with significant amelioration of clinical disease. Similarly, protection in EAE afforded by helminth products is dependent on IL-5 (39). MyD88 is critical for the generation of proinflammatory autoimmune T-cells in response to immunization with autoantigens emulsified with adjuvants such as CFA, which contain TLR ligands (6, 40–42). We previously showed that MOG_35-55_/CFA immunization in MyD88^−/−^ mice induces autoimmune T cells that actively downregulate adoptively transferred encephalitogenic T cells; this suggests that inhibition of MyD88 can generate protective autoimmunity (6). However, since spontaneous EAE develops normally in MyD88^−/−^, PLP-TCR transgenic mice (43), adjuvants may be required to induce MyD88-independent signaling that can regulate autoimmunity. Our current findings support the idea that inhibition of Myd88 during an adjuvant-induced response enables a shift in the T cell cytokine profile. Since the majority of the immune response in MOG_35-55_/CFA-immunized mice is against the foreign antigens of CFA, it is not surprising that systemic MyD88 inhibition influenced the response to PPD, representing many immunodominant antigens of CFA. Mechanistically, MyD88 inhibition may produce a population of partially mature DCs that induce Th2 cells via a default pathway (44, 45). Alternatively, MyD88 inhibition in the context of TLR signaling may lead to the secretion of mediators that directly promote Th2-cell differentiation (46). In any case, therapeutic strategies aimed at MyD88 need to account for the strength and kinetics of the response to the targeted antigen since these parameters influence Th cell development (47, 48).

Several groups have generated small molecule MyD88 inhibitors based on the MyD88 BB-loop seven amino acid peptide (12, 13). These drug candidates have wide-spread potential for use in sepsis, cancer, organ transplantation, and autoimmunity (14, 49, 50). Targeted delivery of MyD88 inhibitors during peaks of Th1 immune reactivity is an attractive option to promote Th2 immunity to self-antigens.

## Ethics Statement

All mouse experiments were approved by the Hebrew University-Hadassah Institutional Animal Care and Use Committee, protocol number 16-14745.

## Author Contributions

GN supervised all experiments and wrote the manuscript together with SD. SD and SC designed and performed all experiments. IC provided conceptual advice and supervised some of the experiments.

## Conflict of Interest Statement

The authors declare that the research was conducted in the absence of any commercial or financial relationships that could be construed as a potential conflict of interest.
