# Supplementary material for: Inhibition of Myeloid Differentiation Factor 88 Reduces Human and Mouse T-Cell Interleukin-17 and IFNγ Production and Ameliorates Experimental Autoimmune Encephalomyelitis Induced in Mice
Source: Front Immunol. 2017 May 29;8:615. doi: 10.3389/fimmu.2017.00615 (PMC5447018; doi:10.3389/fimmu.2017.00615)
Supplement: Supplementary file 1 [file Data_Sheet_1.DOCX]

Supplementary Material

**MyD88 inhibition reduces human and murine T cell IL17 and IFNγ production, and ameliorates adjuvant-induced EAE**

Shira Dishon^1†^, Shmuel J. Cohen^1,2†^, Irun R. Cohen^2^, and Gabriel Nussbaum^1*^

**Supplementary Figure 1.** shRNA reduction in MyD88 expression. Wild-type (WT) and lentivirally transduced THP-1 cells (control and shMyD88) were tested for MyD88 expression by qPCR and WB. The combined results of three independent experiments for each are shown in 1A-B, and a representative WB is shown in C. Reduction in MyD88 expression using this shRNA sequence was also tested in U-937 and HeLa cells (D-E) by qPCR (combined results of three independent experiments).

**Supplementary Figure 2.** MyD88 knock-down does not block the induced expression of HLA-A2 in response to TLR ligands. (A-B) THP-1 cells were differentiated for three days with PMA with or without the addition of 100ng/ml Pam3csk4 + 20ng/ml IFNγ which were added for the final 24 hours. Flow cytometry analysis of HLA-A2 expression is presented. (C) Data is presented as % difference in intensity between each group (ΔMFI). Representative histograms (A, B) and combined data of repetitions from two independent experiments are shown (C).


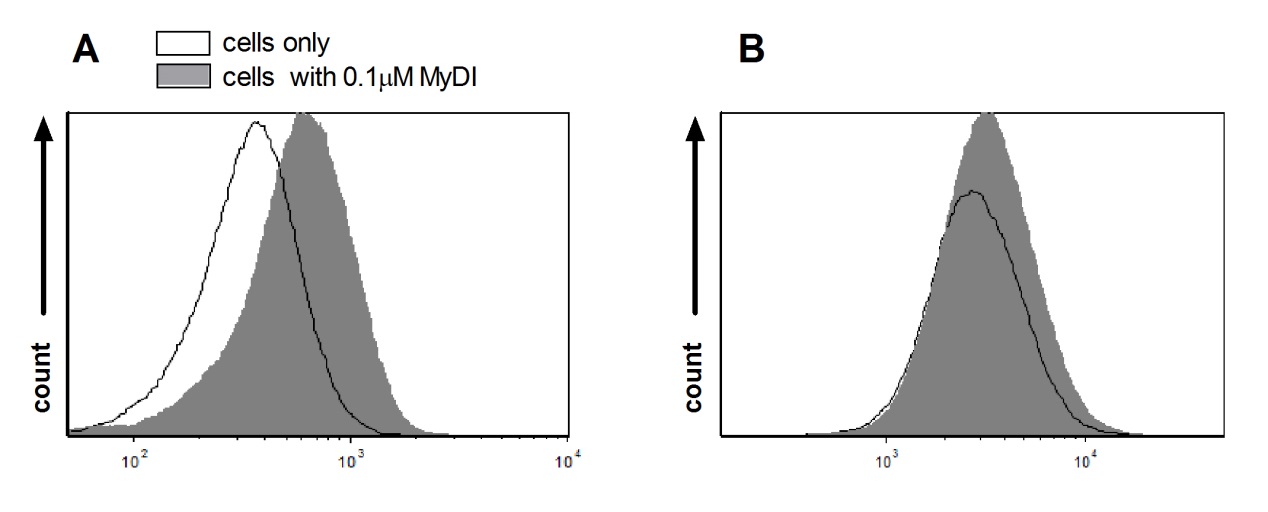


**Supplementary Figure 3.** Penetration of the MyDI peptide to primary human CD4+ T cells vs. THP-1 cells. 0.1uM FITC-labeled MyDI was added to primary human CD4+ T cells or THP-1 cells that were differentiated for three days with PMA. After 3hr incubation, cells were analyzed by flow cytometry. (A) primary human CD4+ T cells, and (B) differentiated THP-1 cells.

**Supplementary Figure 4.** MyDI peptide blocks p65 translocation in response to IL-1b but not TNFα. (A-B) HeLa cells were stimulated with recombinant hTNFα or hIL-1β and NFκB localization was determined with anti-p65 conjugated to rhodamine. The percentage of translocated cells was determined in three independent experiments and summarized together. The number of cells quantified in each experiment is indicated and standard errors are indicated by bars. (A) HeLa cells treated with MyDI and (B) HeLa cells treated with MyDI-sc.

**Supplementary Figure 5.** MyDI peptide blocks hTNFα in response to Pam3csk4. Human PBMCs (A), and THP-1 cells (B), were stimulated with the TLR2 ligand Pam3csK4 (200pg/ml) overnight. 3hr prior stimulation cells were treated with MyDI or MyDI-sc. TNFα levels in the supernatants were determined by ELISA. The results shown are the average of triplicates from one representative experiment of three performed. The 2-tailed t test was used for statistical evaluation.
